# Astrocyte, a Promising Target for Mood Disorder Interventions

**DOI:** 10.3389/fnmol.2019.00136

**Published:** 2019-06-05

**Authors:** Xinyi Zhou, Qian Xiao, Li Xie, Fan Yang, Liping Wang, Jie Tu

**Affiliations:** ^1^Shenzhen Key Lab of Neuropsychiatric Modulation, Guangdong Provincial Key Laboratory of Brain Connectome and Behavior, Brain Cognition and Brain Disease Institute, Shenzhen Institutes of Advanced Technology, Chinese Academy of Sciences, Shenzhen-Hong Kong Institute of Brain Science-Shenzhen Fundamental Research Institutions, Shenzhen, China; ^2^Shenzhen College of Advanced Technology, University of Chinese Academy of Sciences, Beijing, China

**Keywords:** depression, anxiety disorder, neuron, gliotransmitters, astrocytes

## Abstract

Mood disorders have multiple phenotypes and complex underlying biological mechanisms and, as such, there are no effective therapeutic strategies. A review of recent work on the role of astrocytes in mood disorders is thus warranted, which we embark on here. We argue that there is tremendous potential for novel strategies for therapeutic interventions based on the role of astrocytes. Astrocytes are traditionally considered to have supporting roles within the brain, yet emerging evidence has shown that astrocytes have more direct roles in influencing brain function. Notably, evidence from postmortem human brain tissues has highlighted changes in glial cell morphology, density and astrocyte-related biomarkers and genes following mood disorders, indicating astrocyte involvement in mood disorders. Findings from animal models strongly imply that astrocytes not only change astrocyte morphology and physiological characteristics but also influence neural circuits via synapse structure and formation. This review pays particular attention to interactions between astrocytes and neurons and argues that astrocyte dysfunction affects the monoaminergic system, excitatory–inhibitory balance and neurotrophic states of local networks. Together, these studies provide a foundation of knowledge about the exact role of astrocytes in mood disorders. Importantly, we then change the focus from neurons to glial cells and the interactions between the two, so that we can understand newly proposed mechanisms underlying mood disorders, and to identify more diagnostic indicators or effective targets for treatment of these diseases.

## Introduction

Mood disorders are a group of illnesses that describe a serious disturbance in a person’s mood (Sadock and Sadock, 2011), such as major depression disorder (MDD) and bipolar disorder (American Psychiatric Pub Association, 2013), and are a worldwide problem in modern society. According to the World Health Organization (WHO), more than 300 million people are living with depression disorder and this disorder has been ranked as the largest contributor to non-fatal health loss (World Health Organization [WHO], 2017).

Mood disorders lead to substantial personal and social burdens, yet efficacious therapeutic targets for these disorders are currently lacking (Nemeroff and Owens, 2002; Colpo et al., 2018; Boas et al., 2019), due to our relatively poor mechanistic understanding of the neurobiology involved. Determining the cellular mechanisms and neural circuits involved and how they operate is, therefore, an important step to find better therapeutic intervention targets.

Astrocytes, star-shaped non-neuronal cells found in the central nervous system, are traditionally thought to be supporting cells that provide homeostatic control and trophic support within the brain (Lindsay, 1979; Walz and Hertz, 1983; Schousboe et al., 1997; Parpura and Verkhratsky, 2012). Given that a single astrocyte may interact with as many as 100,000 synapses in mice and possibly up to 2,000,000 synapses in humans (Bushong et al., 2002; Oberheim et al., 2009), astrocytes are likely more than simple support cells. We also know that the dysfunction of astrocytes influences synaptic activity; evidence has shown that astrocytes can modulate neuronal circuits and influence behavior (Volterra and Meldolesi, 2005). Astrocytes also exert significant control over synapse formation, adult neurogenesis, and vascular tone (Song et al., 2002; Filosa and Iddings, 2013; Chung et al., 2015). As more and more astrocyte-derived active substances are found, such as glutamate and D-serine, the concept of the “tripartite synapse” has been established (Araque et al., 1999; Perea et al., 2009; Perez-Alvarez and Araque, 2013), which represents the ability of astrocytes to participate in synaptic activity. Based on numerous observations of reduced glial cell numbers from postmortem histopathologic studies of depressed patients, it has been posited that abnormal astrocyte function may contribute to the pathophysiology of mood disorders (Cotter D. R. et al., 2001; Rajkowska and Miguel-Hidalgo, 2007; Hercher et al., 2009).

We review current evidence, mainly on the mechanisms involving mood disorders through which astrocytes are thought to function, with a particular emphasis on depression. We also add evidence from anxiety disorders due to the comorbidity between depression and anxiety disorder (Mineka et al., 1998; Watson, 2005). We first discuss some human studies that have helped clarify astrocyte function in depression before examining some hypotheses that have been proposed to explain the roles that astrocytes play in depression and anxiety disorders. Notably, these multiple hypotheses attempt to explain the same question, and it may be possible to reconcile them rather than discard any. We underscore the utility of a global view when approaching mechanistic questions about depression due to complex diagnostic indicators and widespread biological effects of the disorder. Finally, we discuss some issues arising from astrocyte heterogeneity, such as interspecies differences, subtypes of astrocytes and different interactions between astrocytes and neurons (Oberheim et al., 2009; Haim and Rowitch, 2017; Lin et al., 2017). This heterogeneity should be taken into consideration when studying the relationship between astrocytes and mood disorders. The aim of this review is to promote the idea that astrocytes influence mood disorders and to give a brief view about the current understanding of the possible mechanisms through which astrocytes can be a target for mood disorder interventions.

## Astrocyte Involvement in Mood Disorders: Human Data

Negative emotions, like anxiety and fear, can aid survival by increasing awareness of possible imminent harm. However, mood disorders may occur when negative emotions become persistent, disruptive or inappropriate to the perceived threat. According to the WHO, the number of people suffering from depression increased by more than 18% between 2005 and 2015 (World Health Organization [WHO], 2017). Although there is a lack of effective therapies to treat these mood disorders, there is human evidence showing that abnormalities in glial cells may alter normal brain function and likely contribute to mood disorder development (Rajkowska and Stockmeier, 2013). As such, these human data should not only guide animal model-based research programs but also be used to identify diagnostic indicators.

The first type of evidence connecting astrocytes to mood disorders is through cell counting studies and cell morphology in patients who had mood disorders. In subjects diagnosed with MDD, cell counting studies report that glial cell number and density were decreased in many brain regions, including the anterior cingulate cortex (Cotter D. et al., 2001; Gittins and Harrison, 2011), the dorsolateral prefrontal cortex (PFC) (Cotter et al., 2002), and the amygdala (Bowley et al., 2002), compared to non-psychiatric control subjects. In addition, a decrease of glial cell density has also been observed in subjects diagnosed with bipolar disorder (Grazyna Rajkowska et al., 2001). However, some postmortem work found no change in glia density in the orbitofrontal cortex (Khundakar A. et al., 2011), anterior cingulate cortex (Khundakar A. A. et al., 2011) or hippocampus (Cobb et al., 2013) in subjects that had MMD in life. In addition to changes in glial cell density, an increased glial cell nuclei size has been observed in the dorsolateral PFC in MMD patients (Rajkowska et al., 1999). Hypertrophy of astrocyte cell bodies and processes have also been observed in the anterior cingulate cortex (Torres-Platas et al., 2011). Together, this provides associational evidence of a relationship between mood disorders and abnormal glia pathology.

The second line of evidence from humans relates to altered levels of potentially astrocyte-specific biomarkers in postmortem brain specimens of individuals that had suffered from mood disorders. Low levels of a traditional astrocyte marker, glial fibrillary acidic protein (GFAP), have been found in the hippocampus, PFC, anterior cingulate, and amygdala (Müller et al., 2001; Webster et al., 2001; Altshuler et al., 2010; Gittins and Harrison, 2011).

Interestingly, a consistent finding is that young and mixed age groups of MDD patients have lower GFAP- immunoreactive (IR) astrocyte density in cortical areas than control patients (Öngür et al., 1998; Gittins and Harrison, 2011). However, studies performed on late-onset depression patients (commonly defined as occurring after age 50 or 60) have reported an increase in the density of GFAP-IR astrocytes than younger MDD patients (Khundakar and Thomas, 2009; Paradise et al., 2012). This indicates the astrocyte pathology in cortical areas is different in younger and older patients with depression (Miguel-Hidalgo et al., 2000; Khundakar and Thomas, 2009). Moreover, it is not known whether GFAP simply reflects the astrocytic function and/or whether it is directly associated with the symptoms of mood disorders. Another astrocyte marker, S100B, is a calcium-binding protein predominantly expressed in the cytoplasm that can be secreted to extracellular space and thus be detected in the serum (Gerlach et al., 2006; Andreazza et al., 2007). Mood disorder patients have increased S100B levels (Schroeter et al., 2002), and serum concentration of S100B may be a possible predictor of antidepressant response in patients (Arts et al., 2006; Ambree et al., 2015). Damaged astrocytes release an excess S100B into the serum (Rothermundt et al., 2003), so this should be taken into consideration when increased S100 levels are detected in serum.

A third line of studies from postmortem brain tissue show astrocyte dysfunction of gene transcription and protein expression in patients diagnosed with mood disorders. For example, gene and protein expression of some astrocyte function-related proteins including glutamine synthetase, glutamate transporters, and even gap junction proteins are down regulated in patients with depression (Sequeira et al., 2009; Bernard et al., 2011). Astrocytes convert glutamate into glutamine by glutamine synthetase, so glutamine synthetase and glutamate transporters associated with astrocytes reflect astrocytic function in glutamate transmission (Norenberg and Martinez-Hernandez, 1979; Sonnewald et al., 1997; Anderson and Swanson, 2000). Expression of glutamine synthetase by mRNA is down-regulated in the dorsolateral PFC, premotor cortex and the amygdala of depressed patients (Sequeira et al., 2009) and microarray analysis of specific areas of MDD patient-cerebral cortex show down-regulation of SLC1A2 and SLC1A3, two glial high-affinity glutamate transporters (Choudary et al., 2005). Connexin 30 and connexin 43 are gap junction-forming membrane proteins located on astrocyte endfeet, and dysfunction of the two proteins may alter calcium wave propagation and communication between astrocytes (Blomstrand et al., 1999; Giaume and Theis, 2010). Interestingly, the decreased expression of connexin 30 and connexin 43 has been observed in the dorsolateral PFC of suicide completers with MDD (Blomstrand et al., 1999). It has been reported recently that aquaporin-4 (AQP4), a protein located predominantly in astrocytic endfeet, has lower expression levels in MDD patients compared to non-psychiatric control subjects (Rajkowska et al., 2013). The reduction of AQP4 may influence many astrocytic functions, such as maintenance of the blood brain barrier’s integrity (Nico et al., 2001), glutamate turnover (Zeng et al., 2007), and synaptic plasticity (Li et al., 2012).

Here, we need to bear in mind that studies based on humans, especially postmortem studies, have many limitations, including individual differences and multiple causes of death. For example, the age at which mood disorder onset occurs may influence the number of GFAP-IR astrocytes (Öngür et al., 1998; Khundakar and Thomas, 2009) and postmortem interval may influence the quality of RNA obtained from postmortem brains (Lipska et al., 2006). These limitations make it difficult to generalize results and so, at this stage, appropriate animal models and experimental studies are of equal importance.

## Astrocyte Involvement in Mood Disorders Through Dysfunction of Synapses

A wide body of evidence has shown that depression reshapes brain structures, leading to changes at the level of both synapse and behavior (Christoffel et al., 2011b). Changes in dendritic spines can be roughly divided into three categories: changes in density, in morphology, and in function. Studies from a stress model of depression have reported that spine density is increased or decreased in a region dependent manner; for instance, decreased spine density has been observed in hippocampus CA1 and CA3 cells (Magariños et al., 1997; Qiao et al., 2014), whereas increased spine density has been found in amygdala (Vyas et al., 2006) and NAc (Christoffel et al., 2011a). Spines can be categorized into three subtypes: mushroom, thin and stubby spines, according to their length, the diameter of spine head and the diameter of spine neck (Nimchinsky et al., 2002). Different spine subtypes have different functions and the ratio of these spines can influence neuronal excitability and function (Qiao et al., 2016). Decreased spine volume and surface area have been observed in the brains of stressed rodent models (Radley et al., 2008). Along with changes in spine density and morphology, synapse function also becomes abnormal (Liu and Aghajanian, 2008; Christoffel et al., 2011a). The shift in spine density and morphology may result in a decrease in the number of functional mature spines on neurons and may reflect the dysfunction of synaptic efficacy (Duman and Duman, 2015).

Interestingly, changes in dendritic spines in stress models is not only related to stress stimulation but is also affected by other physiological variables, such as gender and age. Dendritic morphology and spine density of pyramidal neurons in layers II–III of the prelimbic cortex vary with rat gender during recovery from chronic restraint stress (Moench and Wellman, 2017). In addition, stress stimulation leads to dendritic spine loss and changes of spine morphology in prefrontal cortical neurons in young rats but not in middle-aged and aged rats (Bloss et al., 2011). Not all neurons are affected by stress to the same degree; for example, it has been found in rats that a subpopulation of infralimbic neurons in the mPFC that project to the basolateral amygdala are resilient against the effects of stress (Shansky et al., 2009).

Synaptic changes in neurons is a hallmark of depression and astrocytes, as integral components of tripartite synapses, very likely participate in the pathology of depression and anxiety disorder (Bender et al., 2016). Although astrocytes cannot produce action potentials, evidence has shown that astrocytes change morphology and alter the expression of some proteins in response to stimuli (Allen and Barres, 2005). In fact, astrocyte morphology is now known to be very dynamic, with filopodia-like processes moving or growing in the space of only a few minutes (Bernardinelli et al., 2014). Multiple lines of evidence indicate that astrocytes can influence the synaptic plasticity of neurons, not only during synaptogenesis (Christopherson et al., 2005), but also in mature synapses (Jones et al., 2011). There is accumulating evidence that astrocytes remodel synapses, coming from mice strains that are knocked-out or knocked-down for astrocyte-secreted synapse modifying factors [for example, hevin (Singh et al., 2016), SPARC (Jones et al., 2011), and TNF-α (Stellwagen and Malenka, 2006)].

## Astrocytes Influence Mood Disorders Through Interaction With Neurons

Depression can be divided into subtypes according to psychiatric symptoms and is thought to share some biological mechanisms with anxiety disorder. In some cases, depression can arise in people with anxiety disorder and symptoms of depression and abnormal anxiety levels are often observed together. In humans, exposure to stress is a predominant risk factor for depression (Kessler, 1997) and may trigger some susceptible genes that are associated with depression (Caspi et al., 2003; Kaufman et al., 2006). As such, stress paradigms are often adopted in studies investigating depression because of their co-incidence. In this section, the focus is on possible mechanisms of depression and anxiety disorder generally; most studies are from rodent models and we do not distinguish among subtypes unless stated specifically.

### The Physiological Basis by Which Astrocytes Modulate Neurons

Numerous studies have suggested that astrocytes can respond to external signals and release transmitters like glutamate, ATP, D-serine, and lactate, as well as gamma-aminobutyric acid (GABA) (Sahlender et al., 2014; Martín et al., 2015; Machler et al., 2016; Papouin et al., 2017; Tan et al., 2017). Interactions between neurons and astrocytes through gliotransmitters is a possible mechanism in the development and maintaining of mood disorders. Although the mechanism through which astrocytes release these transmitters remains controversial, two hypotheses have been proposed, including vesicular exocytotic release and non-exocytotic release mechanisms.

The vesicular exocytotic release theory holds that astrocytes release gliotransmitters through exocytosis. The existence of synaptic-like vesicles in astrocytes in different brain areas has been observed using electron microscopy (Stranna et al., 2011; Dickens et al., 2017). The SNARE (soluble *N*-ethylmaleimide-sensitive factor attachment protein receptor) complex, which is Ca^2+^dependent, is widely accepted as the main biochemical driver of exocytosis. SNARE complexes in astrocytes are comprised of different subunits to those in neuronal SNARE complexes. The subunit synaptosomal-associated protein 23 (SNAP23) undertakes an analogous role to neuronal SNAP25 (Hepp et al., 1999), and the vesicle-associated membrane protein 3 (VAMP3) subunit has an analogous role to neuronal VAMP2 (Bezzi et al., 2004; Schubert et al., 2011). Two genetic mouse models [the dominant negative (dn) SNARE mouse (Pascual et al., 2005) and the iBot mouse (Slezak et al., 2012)] based on the variant forms of the astrocyte SNARE complex have been used to demonstrate roles that astrocytes play in several physiological and pathological processes (Hines and Haydon, 2013; Nadjar et al., 2013; Turner et al., 2013).

Alternatively, non-exocytotic release mechanisms are also possible. Firstly, there are some astrocyte channels that can mediate gliotransmitter release, such as Bestrophin-1 (BEST1) (Lee et al., 2010; Woo et al., 2012; Oh and Lee, 2017) and astrocyte gap junction hemichannels (Contreras et al., 2002; Stout et al., 2002; Ye et al., 2003). There is evidence that BEST1 is permeable to GABA and glutamate (Lee et al., 2010; Woo et al., 2012; Oh and Lee, 2017), however, studies that focus on structure show that this channel excludes larger molecules such as amino acids (Dickson et al., 2014; Vaisey et al., 2016). The mechanisms for anion selectivity of BEST1 still need to be explored. Secondly, the function of some astrocytic channels and transporters, for example, P2X purinoceptor 7 (P2X7) receptor channels (Virginio et al., 1999; Duan et al., 2003) and astrocytic glutamate re-uptake transporter (Rossi et al., 2000), may change under certain conditions and thereby release gliotransmitters to neurons. Third, exocytotic release itself brings vesicular membranes to the plasma membrane, which may contain channels or transporters that can release gliotransmitters to the surface of the astrocytes, thus leading to non-exocytotic release (Bowser and Khakh, 2007; Parpura et al., 2010).

Although mechanisms that underlie gliotransmission remain controversial, the pathways that astrocytes accept signals from, prior to releasing gliotransmitters, has been widely accepted to be dependent on intracellular Ca^2+^. Infusion of Ca^2+^ buffer solutions in astrocytes can influence synaptic activity (Di Castro et al., 2011; Panatier et al., 2011). What’s more, chelating astrocytic Ca^2+^ via patch clamp through chelators can block the effect that astrocytes have on neurons (Chen et al., 2016; Tan et al., 2017). In addition, fluorescent calcium sensors, Fura-2 and GCaMP6s, have been used to detect changes in astrocyte cytosolic Ca^2+^ and exocytosis-related elevation of Ca^2+^ concentration has been demonstrated (Parpura et al., 1994; Perea et al., 2014; Ma et al., 2016; Tan et al., 2017).

Although the physiological mechanisms whereby astrocytes modulate neurons is not completely determined, many studies have begun to reveal the mechanisms by which astrocytes may contribute to mood disorders.

### The Monoaminergic Hypothesis in Depression and Anxiety Disorder

The momoaminergic hypothesis postulates that depressive symptoms are due to a deficit or imbalance in the central monoaminergic system, which includes serotonergic, dopaminergic, and/or noradrenergic neurotransmission (Tissot, 1975; Syvälahti, 1987). We know that serotonin and noradrenaline play a role in depression and anxiety disorder because of two antidepressant drugs, iproniazid and imipramine. Iproniazid, a monoamine oxidase inhibitor, was first used to treat tuberculosis (Bloch et al., 1954; Ogilvie, 1955), whereas imipramine, a tricyclic antidepressant, was originally developed as an antipsychotic compound to treat schizophrenia (Campbell et al., 1971). Both drugs have been found to improve symptoms of depression, possibly by increasing the efficiency of synaptic monoaminergic neurotransmitters (Smith et al., 1963; Molina et al., 1990; Siris et al., 1994). Because of the existence of the drug-resistant depression (Thase et al., 1992) and drug side effects (Crane, 1956; Saraf et al., 1974), these two drugs are no longer commonly used. Second generation medications, such as serotonin selective reuptake inhibitors (SSRIs) and norepinephrine selective reuptake inhibitors have since been developed and are still widely used today (Nestler et al., 2002).

In the adult rodent brain, many lines of evidence indicate that astrocytes cannot only detect serotonin (5-HT) and noradrenaline (Porter and Mccarthy, 1997) but can also uptake these transmitters. To detect these neurotransmitters, astrocytes express 5-HT receptors, such as 5-HT_1A_ and 5-HT_7_ (Whitaker-Azmitia et al., 1993; Shimizu et al., 1996), and also adrenergic receptors (targets for noradrenaline), such as α1, α2, β1, and β2 (Junker et al., 2002). Moving various neurotransmitters, including monoamines, from the synaptic cleft is one of multiple astrocytic duties and, in order to meet this duty, astrocytes contain many transporters, for instance, glial serotonin transporter (SERT) (Sur et al., 1996) and noradrenaline transporter (NET) (Inazu et al., 2003). SSRIs and tricyclic antidepressants can downregulate the expression of SERT (Inazu et al., 2001), leading to an increase of 5-HT and it is known that the tricyclic antidepressant imipramine decreases the density of astrocytic β1 adrenergic receptors in the rat forebrain (Sapena et al., 1996). This suggests that astrocytes may contribute to depression and anxiety disorder through a deficit in the central monoaminergic system.

### The Breaking of the Excitatory–Inhibitory Balance in Depression and Anxiety Disorder

Excitement and inhibition are two opposing processes that control the activity of neural populations. An imbalance between excitatory and inhibitory neurotransmissions may lead to aberrant functional connectivity patterns within brain circuits. Many clinical imaging studies, such as magnetic resonance spectroscopy (MRS) work (Salvadore et al., 2012; Milak et al., 2016), has shown changes in glutamate and GABA concentrations and activity in patients with depression, suggesting that an imbalance in excitatory and inhibitory neurotransmission may play a role in depression.

**FIGURE 1:**
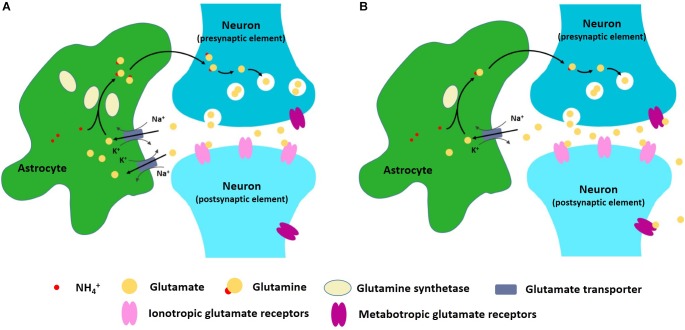
**(A)** Astrocytes can take up, metabolize, and recycle glutamate that is released into the synapses. **(B)** In depression and anxiety disorder conditions, astrocyte dysfunction may lead to excitatory–inhibitory imbalance within neural networks.

One important astrocyte task is to take up, metabolize, and recycle glutamate that is released into the synapses (Magistretti, 2006). When glutamatergic neurons are excited, glutamate is released into the synaptic cleft and bonds to the receptors in the post-synaptic membrane, thus transmitting the signal downstream (Traynelis et al., 2010). The remaining glutamate in the cleft then needs to be removed so that the signal can be stopped. Glutamate removal and recycling is mediated by surrounding astrocytes (Schousboe et al., 2014). The first aspect of astrocyte participation in the excitatory–inhibitory imbalance hypothesis of depression is, therefore, related to deficient glial re-uptake of glutamate. Some proteins, such as glutamine synthetase, are astrocyte-specific and are necessary in the recycling of glutamate (Norenberg, 1979; Rothstein et al., 1994). Reduced expression and content of glial-specific glutamine synthetase and glutamate transporter 1 (GLT1) has been observed in postmortem studies of patients with depression (Choudary et al., 2005), suggesting that glutamate clearance and metabolism are likely impaired in some brain regions. If glutamate re-uptake is blocked, excessive glutamate may stimulate extrasynaptic NMDA receptors, thus promoting cell death (Hardingham and Bading, 2010). Some NMDA receptor antagonists, like ketamine, can produce a rapid antidepressant effect (Newport et al., 2015). Moreover, excessive extrasynaptic glutamate is also taken up by presynaptic metabotropic glutamate receptors and this leads to a reduction in synaptic glutamate transmission (Figure 1) (McEwen et al., 2016). Although we don’t fully understand the mechanism behind astrocyte dysfunction in glutamate re-uptake, inflammation may be a key factor in this (McNally et al., 2008). In patients with depression and anxiety disorders, increased inflammation [as judged by excess levels of inflammatory mediators such as high-sensitivity C-reactive protein level (Danese et al., 2009)] has been detected (Felger, 2018) and inflammation causes impaired astrocytic glutamate uptake (Haroon et al., 2016; Felger, 2018). Astrocytes respond to infection by synthesizing pro-inflammatory and anti-inflammatory cytokines, such as interleukin-1β and tumor necrosis factor-α (TNF-α) (Dantzer et al., 2008). Cytokines such as these stimulate a cascade of inflammatory changes that include activation of proteins such as mitogen-activated protein kinases (MAPK) (Ji et al., 2002; Gorina et al., 2011).

Besides deficient glial re-uptake of glutamate, depression and anxiety are also associated with glutamate receptors, including NMDA and AMPA receptors. Some NMDA receptor antagonists produce antidepressive effects in animal models of depression (Skolnick et al., 1996; Li et al., 2010). A second aspect of astrocyte participation in the excitatory–inhibitory imbalance hypothesis of depression and anxiety disorder is, therefore, related to altered glutamate receptor function. Astrocytes can secrete proteins such as glypicans, and by doing so, recruit additional AMPA receptors to synapses, which will amplify neuronal transmission (Liddelow and Barres, 2015).

In rats, astroglial degeneration in the PFC is a useful depression model (Domin et al., 2014). In addition, 3-((2-Methyl-4-thiazolyl)ethynyl)pyridine (MTEP), a mGluR5 antagonist, may alleviate depression symptoms of the astroglial degeneration model through inhibition of glutamatergic transmission (Domin et al., 2014). Blocking astrocyte-specific GLT1 receptors using pharmacological inhibitors induces depressive-like phenotypes in rats (Bechtholt-Gompf et al., 2010). In summary, astrocyte dysfunction may lead to excitatory–inhibitory imbalance within neural networks, which eventually results in depression and anxiety disorder.

### The Neurotrophic Hypothesis in Depression and Anxiety Disorder

Lower serum levels of neurotrophic factors, such as brain-derived neurotrophic factor (BDNF) are often observed in patients with depression (Pisoni et al., 2018) and increased expression of neurotrophic factors, such as BDNF or glial cell-derived neurotrophic factor (GDNF), have been reported in multiple studies as a response to antidepressant treatment. As a result, the neurotrophic hypothesis of depression was proposed. In addition, evidence that reduced neurotrophic factor levels are tightly linked with neuronal atrophy in certain brain areas in individuals with MDD, such as the PFC and the hippocampus (Duman and Monteggia, 2006), led to the neurotrophic hypothesis of depression. Interestingly, in MDD patients with suicidal ideation, serum BDNF levels are significantly lower than MDD patients with no suicidal ideation (Khan et al., 2019), which suggests that serum BDNF levels may have a complex relationship with MDD symptoms.

Neurotrophic factors can promote neurogenesis, gliogenesis, and synaptic structure remodeling (Koyama, 2015). Astrocytes are a source of these neurotrophic factors (Chen et al., 2006) and so a decrease in such factors may be a mechanism through which astrocytes influence mood disorders, in particular considering that in hippocampus, BDNF overexpression in astrocytes leads to anxiolytic-/antidepressant-like activity in mice (Quesseveur et al., 2013).

Whilst there are different opinions on the mechanisms underlying mood disorders, it is likely that there are multiple factors involved. When searching for the mechanisms by which astrocytes influence mood disorders, a combination of current hypotheses is needed. For example, excessive glutamate may stimulate extrasynaptic NMDA receptors as mentioned above, and the activated extrasynaptic NMDA receptors inhibit the BDNF expression pathway (Hardingham et al., 2002). Additionally, monoamine dopamine induces BDNF upregulation in astrocytes, mainly through β adrenoreceptors (Koppel et al., 2018).

There is also evidence suggesting that astrocytes influence mood disorder through other mechanisms, for example, through gap junctions and hormones. Inhibition of CX43, a main component of astrocytic gap junctions, can lead to depressive-like behavior in rodents (Sun et al., 2012). In addition, the knockout of insulin receptors in astrocytes results in depression-like behavior in mice (Cai et al., 2018). On account of the complexity of depression and anxiety disorders, more detailed mechanisms and effective drug targets should arise following enhancement of understanding of the neurobiology underlying these mood disorders.

## Discussion and Conclusion

Mood disorders are measured by a strong psychological component in humans; they are difficult to quantify and studying their physiopathology remains challenging. However, there are some physical and behavioral symptoms that we can identify, including loss of appetite and abnormal anxiety levels. These are emotional-related behaviors that are thought to be largely preserved during evolution and can be identified across species (Darwin, 1998; Anderson and Adolphs, 2014; Janak and Tye, 2015). Thus, with appropriate methods, we can use animal models to study astrocytic mechanisms involved in mood disorders. We should also take differences across species into consideration because astrocyte number and size increases reflecting brain size and cognitive capabilities (Allen, 2014; Stogsdill and Eroglu, 2017). Rodents are one of the most widely used animal models and there are many differences in rodent astrocytes compared to human astrocytes. Astrocytes in human brains have larger populations, signal faster, are bigger, and are more structurally complex than those of rodents (Oberheim et al., 2009). Gene expression studies have also identified novel human-specific astrocytes (Zhang et al., 2016). Interestingly, transplanting human glial progenitors into the adult mouse brain enhances synaptic plasticity and behavioral learning (Han et al., 2013).

With more and more studies focusing on astrocytes, our understanding is developing. Yet, more problems arise when studying and interpreting astrocyte function. One is related to different astrocyte populations in different brain regions or even within the same region. A recent study identified five distinct astrocyte subpopulations, and these populations differentially support synaptogenesis between neurons (Lin et al., 2017). In glial scar area, reactive astrocytes have also shown heterogeneity, findings that have contributed to debate about whether or not glial scar aids CNS regeneration (Adams and Gallo, 2018). Even within one pathological or physiological condition, astrocytes may be able to play different roles in different brain regions or show heterogenetic influences on the same neurons (Haim and Rowitch, 2017; Martin-Fernandez et al., 2017). Deciphering the diversity of astrocytes and elucidating their functions *in vivo* is an important next step. These heterogenetic astrocytes may add more complexity to studies but should also help understand the complex mechanisms behind mood disorders and confirm the view that astrocytes are more than just “glue.”

## Author Contributions

XZ, QX, and JT conceived of and wrote this manuscript. LX, FY, and LW provided suggestions.

## Conflict of Interest Statement

The authors declare that the research was conducted in the absence of any commercial or financial relationships that could be construed as a potential conflict of interest.
